# Editorial: Plant reproduction under environmental stress

**DOI:** 10.3389/fpls.2024.1369070

**Published:** 2024-02-26

**Authors:** Kevin Begcy, Marta A. Mendes, Nico De Storme

**Affiliations:** ^1^ Environmental Horticulture Department, University of Florida, Gainesville, FL, United States; ^2^ Plant Molecular and Cellular Biology Graduate Program, University of Florida, Gainesville, FL, United States; ^3^ Dipartimento di Bioscienze, Università degli Studi di Milano, Milan, Italy; ^4^ Laboratory for Plant Genetics and Crop Improvement, Division of Crop Biotechnics, Department of Biosystems, Heverlee, KU Leuven, Belgium

**Keywords:** flowering transition, ovule, pollen, seeds, stress responses, heat, cold, epigenetics

Reproductive development is an essential phase during the life cycle of flowering plants. It ensures preservation of karyotypic configuration and genomic stability, and additionally creates novel genetic variation in the offspring. In the current context of global warming and climate change, with plants being more intensively challenged by extreme environmental conditions, insights into the sensitivity of the molecular and physiological responses of the reproductive pathway to external stresses are highly relevant for both plant evolution and crop productivity. In this Research Topic, we present the impact of low and high temperatures as well as low light conditions on plant reproduction.

## Impact of low temperatures on plant reproduction

For some crops, low temperatures are a serious threat that results in drastic yield decline. Using tomato (*Solanum lycopersicum*) as model plant, Yang et al. explored whether N6-methyladenosine (m^6^A) regulates pollen development under low temperature stress and found that this condition affects pollen development at the tetrad stage, causing pollen sterility, characterized by changes in tapetum development and exine deposition. m^6^A levels in anthers were decreased at both the tetrad and the uninucleate stages under low temperature stress compared to control conditions. Further transcriptome-wide detection of m^6^A methylation levels in anthers at the tetrad stage under low temperature conditions showed that cold conditions affect m^6^A levels in many predominantly downregulated mRNA transcripts. These differentially m^6^A enriched transcripts under low temperature conditions were mainly related to ATP-binding pathways, adenosine triphosphatase activity, and lipid metabolism. Interestingly, *SlABCG31*, an ATP-binding transcript predominantly expressed in anthers, was proposed to participate in the regulation of abscisic acid (ABA), as its significant m^6^A upregulation was correlated with higher ABA levels in anthers. These interesting findings suggest that the decrease in m^6^A levels in response to low temperatures results in the altered transcription of many pollen development-related genes impacting anther ABA levels, leading to pollen sterility.

Another exciting story comes from a study in which the authors explored the impact of low temperatures on the transition from the vegetative to the reproductive phase (Nasim et al.). This critical developmental transition is primarily regulated by the expression of *FLOWERING LOCUS C* (*FLC*) and other clade-related genes controlled by Polymerase II-Associated Factor 1 Complex (PAF1C - *CELL DIVISION CYCLE73/PLANT HOMOLOGOUS TO PARAFIBROMIN*, *VERNALIZATION INDEPENDENCE2* (*VIP2*)/*EARLY FLOWERING7* (*ELF7*), *VIP3*, *VIP4*, *VIP5*, and *VIP6/ELF8*). In *Arabidopsis thaliana* T-DNA PAF1C-deficient mutant lines, strong early flowering was observed as temperature was decreased. Since changes in epigenetic status have been shown to be critical for the stress response during reproductive development (Begcy and Dresselhaus, 2018), Nasim et al. analyzed the levels of H3K27me3 and H3K4me3/H3K36me3 marks on *FLC* and the *FLC*-clade genes. Under low temperature stress, *FLC* and *FLC*-clade genes showed significantly increased enrichment of H3K27me3 and significantly reduced enrichment of H3K4me3 marks in PAF1C mutants, which is consistent with their transcriptional downregulation. These results show that PAF1C is required to maintain active histone H3K4me3 marks while preventing the deposition of H3K27me3 repressive marks at the *FLC* and *FLC*-clade genes, thereby maintaining their active transcription.

Another aspect covered in this Research Topic is the effect of low temperatures during the bloom period in grapes and their impact on fruit set. Keller et al. manipulated the inflorescence temperature of field-grown Cabernet Sauvignon grapevines during the bloom period. Low temperatures resulted in decreased fruit set, as indicated mainly by the lowest berry number per cluster, as well as seed and berry weight at harvest. At the biochemical level, low temperatures slightly decreased fruit soluble solids and pH, and increased titratable acidity, but did not affect color density. In summary, these results suggest that low temperatures not only decrease the proportion of flowers that set fruit but also limit the sink strength of the berries that do develop after fruit set.

## Impact of heat stress on plant reproduction

High temperatures caused by climate change are negatively impacting reproductive success in many plant species (De Storme and Geelen, 2014; Chen et al., 2016; Begcy et al., 2019). An understanding of how plants try to overcome heat stress during their reproductive phase is fundamental to the effective design of strategies to improve agricultural productivity. In a well-considered review paper, Resentini et al. provided a general overview of the molecular mechanisms underlying heat stress resilience during reproductive development through comparison of a dicotyledonous and a monocotyledonous model system, *Arabidopsis* and *Oryza sativa.* The pollen and the ovule are two reproductive organs whose development is highly susceptible to heat stress. The authors describe four main strands of impact when temperatures are increased during normal development in these two organs: (1) morphological alteration of these tissues, including the anthers and pistils, impacting reproductive success; (2) inhibition of male and female gametophyte development; (3) inhibition of pollen development; and (4) limitation of pollen tube growth. These aspects highlight some of the weakest developmental organs during plant reproduction. Two important additional aspects, summarized by Resentini et al., are recent advances in understanding of the cellular responses to heat stress, represented by the induction of heat shock proteins and calcium signaling. A solid amount of data indicates that heat stress affects plant Ca^2+^ channels, inducing a transient increase in cytosolic Ca^2+^ concentration. Cytosolic Ca^2+^ ion acts as a second messenger that triggers specific cellular responses, including heat shock factors and heat shock proteins. These large groups of transcription factors and chaperones have been used to develop heat resilient crops (ul Haq et al., 2019; Kim et al., 2021).

In cucumber (*Cucumis sativus* L.), high temperature stress always results in sterility at the reproductive stage (Chen et al.). To elucidate the nature of this susceptibility, Chen et al. performed cross-section analysis of anther development in cucumber at high temperatures. The authors observed tapetum abnormality, abnormal microspore, and microspore degeneration in stressed anthers. A high rate of pollen grain abortion and a significant reduction in pollen fertility were also demonstrated. When antioxidant enzymatic activity was measured, including peroxidase, malondialdehyde, catalase, and superoxide dismutase, extremely high levels were found in heat-stressed anthers, which indicates a stressed physiological status. Interestingly, transcriptional analysis showed that carbohydrates and many saccharides and starch synthase-related genes, such as invertase, sucrose synthase, and starch-branching enzymes, were misregulated under exposure to increased temperatures, which represents a common stress response in many plant species, as shown in maize, cotton, and Arabidopsis (Begcy et al., 2019; Masoomi-Aladizgeh et al., 2021; Seydel et al., 2022).

Using a combination of invasive and non-invasive techniques to investigate floret development in barley (*Hordeum vulgare*), Callens et al. showed that while female reproductive organs are less susceptible to heat stress, male reproductive organs are severely impacted. A practical conclusion of this study was that the timing of stress relative to reproductive development has a significant impact on fertility in a cultivar-dependent manner. Immunolabelling of cell wall components in anthers and ovules of heat-stressed barley plants showed that anther cell wall components collapsed (including 1,3;1,4-β-glucan, de-esterified homogalacturonan, and cellulose). The immunolabelling of ovule sections showed that the embryo sac was present in all cases, but there seemed to be fewer cell layers between the embryo sac and the integument. However, no obvious irregularities in the morphology of the embryo sac were identified.

In tomato, the meiosis to early microspore stage of pollen development was found to be the most sensitive to heat stress, and only three days of heat exposure during this developmental window was sufficient to significantly reduce pollen viability at the flower anthesis stage. Cytological analysis showed that abnormalities in pollen development could first be observed after pollen mitosis I, while no deviations in tapetum development were observed. At the transcriptional and biochemical level, pollen development suffered from tapetal endoplasmic reticulum stress (Xu et al.).

Heat stress during seed development is a significant factor in reducing seed size and yield. Using *Camelina sativa*, Nadakuduti et al. explored the impact of high temperatures on fatty acids. Striking differences in the qualitative and quantitative levels of fatty acids were detected. A decrease in percentage polyunsaturated fatty acids and an increase in percentage monounsaturated accumulation of fatty acids was measured under heat stress conditions. The authors suggested that fatty acid biosynthesis shifted to monounsaturated fatty acid synthesis to protect cell function and embryo development by maintaining membrane stability and preventing fatty acid oxidation.

Jansma et al. used a *35S:nahG* tomato line, which has low levels of the stress response-associated hormone salicylic acid, to investigate whether this hormone improves pollen thermotolerance to high temperatures or negatively reduces the investment of resources in reproductive development. Low levels of salicylic acid were found to increase pollen viability under heat stress conditions by enhancing jasmonic acid signaling in developing anthers. These results open the door for alternative pathways within the current biotechnological and breeding strategies that are implemented to ameliorate stress responses.

## Impact of low light on plant reproduction

Aiming to understand alfalfa (*Medicago sativa* L.) development during reproduction under shading conditions, Quin et al. studied flowering phenology, pollen viability, stigma receptivity, and seed quality. In general, low light conditions delayed flowering phenology, shortened the flowering stage, and significantly reduced pollen viability, stigma receptivity, number of flowers, and quantity and quality of seeds. Interestingly, since alfalfa is a forage crop used in intercropping or rotation ecosystems, low light conditions were beneficial in maintaining a high aboveground biomass (Qin et al.). This is likely due to the regulation of the accumulation and distribution of assimilates between the vegetative and reproductive organs.

## Author contributions

KB: Conceptualization, Writing – original draft, Writing – review & editing. MM: Writing – review & editing. NS: Writing – review & editing.
